# Risk Factors Associated with Adverse Events Following COVID-19 Vaccination in Children Aged 5–11 Years: A Real-World Observational Study

**DOI:** 10.3390/children13091160

**Published:** 2026-08-28

**Authors:** Mª Ángeles Rivas Paterna, Carla Pérez Ingidua, Ana B. Rivas Paterna, Belén Joyanes Abancens, Esther Aleo Luján, Emilio Vargas-Castrillón

**Affiliations:** 1 Unidad de Cuidados Intensivos Pediátricos, Hospital Clínico San Carlos, Calle Martín Lagos s/n, 28040 Madrid, Spain; mangeles.rivas@salud.madrid.org (M.Á.R.P.); belen.joyanes@salud.madrid.org (B.J.A.); 2 Unidad de Recuperación Postanestésica, Hospital Clínico San Carlos, Calle Martín Lagos s/n, 28040 Madrid, Spain; 3 Departamento de Enfermería, Facultad de Enfermería, Fisioterapia y Podología, Universidad Complutense de Madrid, Avenida Complutense s/n, 28040 Madrid, Spain; carlap05@ucm.es; 4 Servicio de Farmacología Clínica, Hospital Clínico San Carlos, Calle Martín Lagos s/n, 28040 Madrid, Spain; emilio.vargas@salud.madrid.org; 5 Instituto de Investigación del Hospital Clínico San Carlos (IdISSC), Calle Martín Lagos s/n, 28040 Madrid, Spain; 6 Departamento de Pediatría y Salud Pública, Facultad de Medicina, Universidad Complutense de Madrid, Avenida Complutense s/n, 28040 Madrid, Spain; 7 Hospital Infantil Universitario Niño Jesús, Av. Menendez Pelayo, 65, Retiro, 28009 Madrid, Spain; esther.aleo@salud.madrid.org; 8 Departamento de Farmacología y Toxicología, Facultad de Medicina, Universidad Complutense de Madrid, Avenida Complutense s/n, 28040 Madrid, Spain

**Keywords:** COVID-19 vaccine, children, adverse events, SARS-CoV-2, pediatric vaccination, comorbidity, pharmacovigilance

## Abstract

**Background**: COVID-19 vaccination has demonstrated a favorable safety profile in children; however, factors associated with the occurrence of adverse events (AEs) following immunization remain insufficiently characterized. This study aimed to identify the demographic and clinical factors associated with AEs following COVID-19 vaccination in children aged 5–11 years. **Methods**: We conducted an ambispective observational cohort study in Madrid, Spain, including children aged 5–11 years vaccinated with the 10 µg formulation of BNT162b2 (Comirnaty^®^, Pfizer-BioNTech). Data were obtained from vaccination registries, electronic health records, and structured telephone interviews with parents or legal guardians. Demographic characteristics, anthropometric variables, previous SARS-CoV-2 infection, medical history, and concomitant medication use were evaluated. Univariable analyses and multivariable logistic regression were performed to identify factors independently associated with the occurrence of suspected AEs. **Results:** A total of 2026 children were included. Suspected AEs were reported in 759 participants (37.5%). In univariable analyses, greater weight, height, pre-existing medical conditions, concomitant medication use, clinically vulnerable status, and previous SARS-CoV-2 infection were significantly associated with AEs. In the multivariable model, previous SARS-CoV-2 infection (OR 4.07, 95% CI 3.13–5.30), number of concomitant medications (OR 7.77, 95% CI 5.22–11.56), number of pre-existing medical conditions (OR 1.86, 95% CI 1.62–2.14), and height (OR 1.03, 95% CI 1.01–1.04) remained independently associated with AEs. Age, sex, body mass index, and clinically vulnerable status were not independently associated with AE occurrence. **Conclusions**: In this large real-world pediatric cohort, previous SARS-CoV-2 infection, comorbidity burden, and concomitant medication use were independently associated with adverse events following COVID-19 vaccination. These findings suggest that underlying clinical complexity may be a more important determinant of post-vaccination reactogenicity than demographic characteristics and support the value of individualized pre-vaccination assessment in pediatric populations.

## 1. Introduction

In December 2021, Spain implemented a COVID-19 vaccination program for children aged 5–11 years using the 10 µg formulation of the BNT162b2 vaccine (Comirnaty^®^, Pfizer-BioNTech). Vaccination was recommended for all children in this age group, with initial prioritization of those at increased risk of severe COVID-19.

Since then, recommendations for COVID-19 vaccination have evolved in response to changes in the epidemiology of SARS-CoV-2 infection, increasing population immunity, and the widespread availability of effective vaccines [1]. Although both SARS-CoV-2 circulation and the severity of COVID-19 have decreased substantially in recent years, the virus continues to circulate globally without a clearly defined seasonal pattern. In the pediatric population, infection rates remain relatively high, particularly among infants younger than one year of age [2].

Current recommendations from the World Health Organization (WHO) advocate COVID-19 vaccination for children aged 6 months to 12 years with moderate or severe immunocompromising conditions and suggest vaccination for children with significant underlying medical conditions and previously unvaccinated healthy children aged 6 months to 2 years [3]. Similarly, recommendations from the Centers for Disease Control and Prevention (CDC) and the Advisory Committee on Immunization Practices (ACIP) support COVID-19 vaccination for individuals aged ≥ 6 months, emphasizing the greatest benefit among those at increased risk of severe disease [4]. In Spain, the Advisory Committee on Vaccines of the Spanish Association of Pediatrics (CAV-AEP) currently recommends vaccination for children aged 6 months to 11 years with conditions associated with a higher risk of severe COVID-19, including selected immunodeficiencies, hematopoietic stem cell transplant recipients, and severe chronic respiratory, cardiovascular, neurological, or neuromuscular disorders. Vaccination is also recommended for household contacts of vulnerable individuals and for children living in institutional or long-term care settings [2].

These recommendations encompass a highly heterogeneous pediatric population, including otherwise healthy children of different ages and developmental stages, as well as children with diverse chronic medical conditions affecting multiple organ systems. Differences in age, body composition, underlying diseases, and concomitant treatments may influence vaccine responses and the occurrence of adverse events (AEs) following immunization.

Reported COVID-19 vaccination coverage among children aged 5–11 years varies considerably across countries. Spain has achieved one of the highest vaccination rates worldwide, with approximately 57.3% of children in this age group receiving at least one vaccine dose [5]. Nevertheless, a substantial proportion of eligible children remain unvaccinated. Among the factors contributing to vaccine hesitancy, concerns regarding vaccine safety and the possibility of AEs remain particularly relevant for parents and caregivers.

Although the safety profile of COVID-19 vaccines has been extensively demonstrated in clinical trials and post-marketing surveillance studies, reactogenic AEs such as injection-site pain, fever, fatigue, headache, and myalgia are commonly reported following vaccination. These events are generally mild and self-limiting but may influence parental acceptance of vaccination and contribute to vaccine hesitancy [6]. Furthermore, reactogenicity varies substantially between individuals, suggesting that host-related factors may play a role in determining the occurrence and severity of AEs following immunization. Despite widespread pediatric COVID-19 vaccination campaigns worldwide, evidence regarding factors associated with adverse events in children remains limited. A better understanding of demographic and clinical factors associated with AEs could help identify subgroups at increased risk, improve vaccine counseling, strengthen confidence among families and healthcare professionals, and support evidence-based vaccination strategies aimed at increasing vaccine uptake and reducing disease-related morbidity.

In a previous analysis conducted in the same cohort [7], we characterized the frequency, severity, and clinical features of adverse events following BNT162b2 vaccination and observed an overall favorable safety profile, with predominantly mild and self-limiting reactions. However, that study was not designed to identify the demographic and clinical factors associated with the occurrence of adverse events. The present analysis extends those findings by specifically evaluating potential predictors of adverse events in vaccinated children. The specific aim of this study was to identify the demographic and clinical factors associated with the occurrence of adverse events following COVID-19 vaccination in a pediatric population.

## 2. Materials and Methods

To achieve this objective, we conducted an ambispective observational cohort study involving children vaccinated against COVID-19 in Madrid, Spain. Retrospective data were collected from the patients’ medical records, while prospective data were obtained through structured telephone interviews conducted with parents or legal guardians who provided informed consent [7]. The sample included patients of any sex aged 5–11 years. Given the observational and real-world nature of the study, no formal a priori sample size or power calculation was performed. Instead, we aimed to include all potentially eligible children vaccinated during the study period for whom contact information was available, parental/legal guardian consent was obtained, and sufficient clinical information was recorded.

The present study is a secondary analysis of a previously described cohort of children vaccinated against COVID-19 [7]. The methodology used for participant recruitment, follow-up procedures, and adverse event identification has been reported previously [7]. The current analysis focuses specifically on factors associated with the occurrence of adverse events. Briefly, events were clinically classified according to their presentation, including local reactions, systemic reactions, and other organ-specific events. Timing was assessed according to the interval between vaccine administration and symptom onset, including immediate events identified during the post-vaccination observation period and subsequent events reported during follow-up. Severity was classified according to Venulet criteria as mild, moderate, or severe, and causality was assessed using the Karch–Lasagna algorithm and categorized as unrelated, conditional, possible, probable, or related following the procedures reported in the original cohort study [7].

Participant recruitment and follow-up procedures have been described in detail previously [7]. Briefly, after the administration of the first dose of vaccine, the research team contacted (by telephone) the parents/guardians of the patient to obtain verbal informed consent for the study, and if consent was given, they conducted a semi-structured interview to determine whether the child had experienced any AEs and to obtain details on any such events. If the family could not be reached, the call was repeated up to three times on two different days. Telephone follow-ups were also conducted after the second dose, at one month, and six months post vaccination, to identify potential AEs in the medium and long term. In order to compile information relating to possible risk factors, the patients’ full medical history was reviewed via the HORUS system, which includes records for primary care, specialty care, and other health services, such as the 112 emergency medical services line. If additional information was needed, the team made another call to the parents and healthcare professionals who had reported the event. Data on variables that would allow the investigation of possible risk factors associated with the occurrence of AEs (age, sex, body weight, height, body mass index (BMI), medical history, and pharmacological treatments) were standardized, categorized, and recorded in an electronic case report form (eCRF) designed specifically for this purpose [7]. All the data were recorded using the REDCap 16.1.4 (Research Electronic Data Capture) web application [8].

To avoid memory bias in identifying short-term AEs, it was determined that telephone follow-ups should be conducted on the third day after first and second dose administration, as well as one month and six months later for short- and medium-term AEs. These follow-ups could be conducted up to one week after the recommended date. AEs were collected after both vaccine doses [7].

Data collection was performed by healthcare professionals with expertise in clinical pharmacology and/or pediatrics, following the procedure previously reported [7].

For the purposes of this study, we applied the World Health Organization (WHO) definition of a suspected adverse event as a “noxious, unintended, and undesired response to a medicine that occurs at doses normally used in humans for prophylaxis, diagnosis, or therapy, or for the modification of physiological function.” [9] However, all potentially vaccine-related events were subsequently reviewed and clinically characterized by trained healthcare professionals, as previously described [7].

For the present risk-factor analysis, the primary outcome was defined as the occurrence of at least one suspected AE after COVID-19 vaccination, regardless of clinical subtype, severity, timing, or causality category. Therefore, the outcome was dichotomized as presence or absence of ≥1 suspected AE.

The clinical and pharmacological variables were derived from the original cohort database [7], and to standardize the study variables, the following definitions were used:

- Previous COVID-19 infection was defined as documented SARS-CoV-2 infection occurring before the second dose of vaccination.
- Medical history (antecedents): Each condition was counted as a single medical condition. Multiple diagnoses in the same child were recorded independently and summed to calculate each patient’s comorbidity burden. Medical episodes were grouped according to the main affected organ system.
- Concomitant medication was defined as any medication administered before vaccine administration on the day of vaccination, regardless of whether it was used as regular or acute treatment.
- Chronic treatment was defined as the use of the same medication for at least 3 consecutive months before vaccination.
- Polypharmacy was defined as the concurrent use of three or more medications.
- Drugs were classified according to the Anatomical Therapeutic Chemical (ATC) Classification System at the first level.
- The clinically vulnerable population was defined according to current CAV-AEP recommendations and included children with conditions associated with an increased risk of severe COVID-19 [2].

All the collected data were exported in a compatible format for subsequent analysis with the software package SPSS, version 30.0.

Continuous variables were summarized as means and standard deviations (SDs), whereas categorical variables were described using frequencies and percentages (%). The association between demographic and clinical characteristics and the occurrence of suspected AEs was explored using bivariable analyses. Differences in continuous variables (age, weight, height, and body mass index) between children with and without suspected AEs were evaluated using independent-samples Student’s *t*-tests. Associations between categorical variables and suspected AEs were assessed using Pearson’s chi-square test or Fisher’s exact test when expected cell frequencies were low. Odds ratios (ORs) and their corresponding 95% confidence intervals (95% CIs) were calculated to quantify the strength of the associations. For variables representing counts, including the number of pre-existing medical conditions and the number of concomitant medications, logistic regression analyses were performed to estimate the increase in the risk of AEs associated with each additional condition or medication. Variables showing statistical significance in the univariable analysis and considered clinically relevant were subsequently entered into a multivariable logistic regression model to identify independent factors associated with suspected AEs. Collinearity among candidate predictors was assessed using the variance inflation factor (VIF), and model discrimination was evaluated using the area under the receiver operating characteristic curve (AUC-ROC). A tolerance of <0.20 or a VIF of >5 was considered indicative of significant collinearity. For all tests, we considered a *p*-value of 0.05 or less to be statistically significant.

No imputation of missing data was performed. Only participants with complete information for the variables included in the analysis were retained in the final analytical cohort. Children with insufficient clinical information in their medical records were excluded from the analysis.

This study adhered to the principles of the Declaration of Helsinki and Royal Decree 957/2020 regulating observational studies involving the use of medicines in human subjects. In addition, data collection and handling adhered to Organic Law 3/2018 of December 5 on the Protection of Personal Data and Guarantee of Digital Rights.

The Ethics Committee of the hospital approved the final version of the study (Code: 22/096-O_M_SP) on 24 February 2022, which was registered in the Spanish Register of Clinical Trials (0020-2022-OBS), and we obtained verbal parental informed consent for all participants. This study was monitored by the Clinical Trial Unit UICEC-Hospital Clínico San Carlos [7].

## 3. Results

The source cohort and initial participant flow have been reported previously [7]. Briefly, 3677 children were vaccinated, contact information was available for 2914, 480 could not be reached, and 308 parents declined participation, resulting in an original cohort of 2126 children. For the present secondary analysis, an additional 100 participants were excluded because of insufficient clinical information, yielding a final analytical sample of 2026 children.

**Figure 1:**
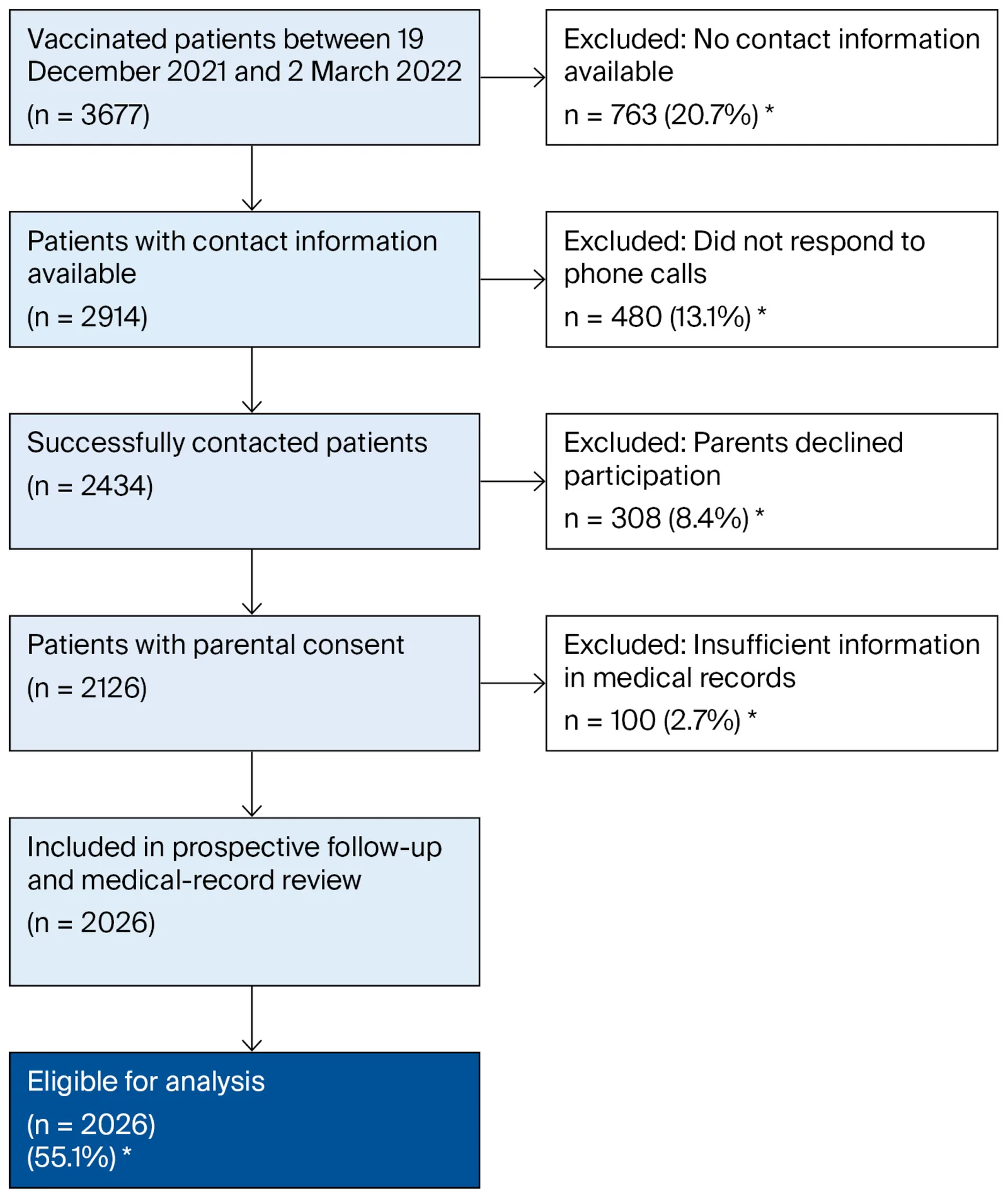
Flowchart showing the selection of vaccinated patients included in the prospective follow-up and medical record review. Of the 3677 vaccinated patients, 2026 were ultimately eligible for the final analysis. * Percentages are calculated relative to the initial number of vaccinated patients (n = 3677).

All children received Comirnaty^®^ 10 micrograms (Figure 1).

Demographic and clinical variables (age, weight, height, body mass index (BMI), sex, concomitant medication, and medical history) were analyzed to determine their relationship with the occurrence of AEs. Pre-existing medical conditions and the drugs administered were categorized in order to identify groups vulnerable to the occurrence of AEs. The demographic characteristics of the original cohort have been reported previously [7]. After excluding participants with insufficient clinical information, the final analytical sample included 2026 children between 5 and 11 years old, with a mean age of 109.5 months (9.1 years) (standard deviation: 25.9 months). A total of 52.2% were male (Table 1).

Participants had a low comorbidity burden: a mean of 0.4 participants recorded pre-existing medical conditions (median: 0; range: 0–7) and were exposed to concomitant medications, averaging 0.1 (median: 0; range: 0–4). Most individuals reported no comorbidities (72.8%) and did not receive concomitant medication (88.2%). Due to their medical history, 127 children were exposed to chronic treatment (6.3%), and polypharmacy was rare (0.5%). A history of COVID 19 was present in 19.3% of the population. Notably, 37.5% of participants experienced at least one AE.

### 3.1. Age

The mean age in the group with no suspected AEs was 107.6 months (8.9 years), and in the group with a suspected AE, it was 108.2 months (9.0 years): a mean difference of −0.6 (95% CI = −2.8; 1.6), Cohen’s d = −0.024. No significant differences in age were found between those who developed AEs and those who did not (*p* = 0.591).

When analyzing age by age group, no statistically significant association was observed between age groups and suspected AEs, χ^2^(2, N = 2026) = 3.6, *p* = 0.164. The proportion of patients with suspected AEs was 34.0% in early middle childhood (5–7 years), 37.1% in middle childhood (7–9 years), and 39.2% in late childhood (9–11 years).

Although a slight increase in the frequency of suspected AEs was observed with age, the differences did not reach statistical significance; therefore, categorized age did not show a relevant association with suspected AEs in the sample analyzed.

### 3.2. Weight

Patients with AEs had a significantly higher weight (32.8 kg) than those who did not develop them (29.9 kg), with a mean difference of −2.9 kg (95% CI: −3.8 to −2.1; *p* < 0.001), Cohen’s d = −0.322. Higher body weight was significantly associated with an increased likelihood of adverse events (small-to-moderate effect).

### 3.3. Height

Patients with AEs were significantly taller (135.4 cm) than those without AEs (130.0 cm), with a mean difference of −5.4 cm (95% CI: −6.6 to −4.1; *p* < 0.001), Cohen’s d = −0.400. Greater height was also associated with an increased likelihood of AEs (moderate effect).

### 3.4. Body Mass Index

Children who experienced AEs had a slightly higher BMI (17.6) than those who did not (17.4); however, this difference was not statistically significant (*p* = 0.185).

Among the three variables analyzed in relation to anthropometric parameters, height and weight showed the strongest association with AE occurrence.

### 3.5. Gender

Of the children who experienced AEs, 372 (49%) were female and 387 (51%) were male. Among all vaccinated girls (N = 969), 372 (38.4%) experienced at least one AE; among all boys (1057), suspected AEs were reported in 36.6% of cases (N = 387). No significant association was observed between sex and the occurrence of AEs (χ^2^ = 0.4; *p* = 0.681).

### 3.6. Concomitant Medication

The use of medication was also significantly associated with AEs (χ^2^ = 286.1; *p* < 0.001). Children receiving concomitant medication before vaccination on the day of immunization experienced AEs more frequently than those not receiving medication (87.1% vs. 30.8%). The OR was 15.1 (95% CI: 10.2–22.4).

When chronic treatments were analyzed separately, a statistically significant association was observed between the use of regular (chronic) medication and AEs, χ^2^ = 73.98, *p* < 0.001. Furthermore, the presence of regular medication was associated with a higher probability of suspected AEs (OR = 5.1; 95% CI: 3.4–7.6). It should be noted that all patients receiving long-term medication had at least one pre-existing medical condition.

A statistically significant association was observed between polypharmacy (≥3 medications on the day of vaccination) and the occurrence of AEs (*p* = 0.001). Suspected AEs were more common in patients with polypharmacy (90.0%) than in those without it (37.2%) (OR = 15.2; 95% CI: 1.9–120.1). The results should be interpreted with caution due to the small size of the group with polypharmacy.

The number of administered drugs showed significant differences between the groups (Table 2). Concomitant medication use was more frequent among children with suspected AEs than among those without suspected AEs. While 72.6% of children with suspected AEs were not receiving concomitant medication, this proportion was 97.6% among children without suspected AEs. Conversely, exposure to at least one concomitant medication was observed in 27.4% of children with suspected AEs compared with 2.4% of those without it.

When the association between drug class and AEs was analyzed, drugs for the musculoskeletal, nervous, or respiratory systems were the three most frequently involved (Table 3). The full ATC-stratified analysis is presented in Supplementary Table S1.

### 3.7. Medical History

Medical history was a factor strongly associated with AEs (χ^2^ = 288.2; *p* < 0.001). Patients with pre-existing medical conditions had AEs in 67.3% of cases, compared to 26.3% of those without them. The OR was 5.8 (95% CI: 4.7–7.1). A significant association was observed between previous allergies and AEs (χ^2^ = 143.9; *p* < 0.001). Children with allergies had a higher risk of AE (70.4%) than non-allergic children (32.4%). The OR was 4.9 (95% CI: 3.7–6.5).

Patients without suspected AEs had an average of 0.22 pre-existing medical conditions, whilst those with suspected AEs had an average of 0.82 (t = −15.3; *p* < 0.001).

A greater number of pre-existing medical conditions was associated with a higher probability of suspected AEs (Table 4). While 85.8% of children without suspected AEs had no recorded comorbidities, this proportion decreased to 51.3% among children with suspected AEs. Conversely, 48.7% of children with suspected AEs had at least one antecedent, compared with 14.2% of those without suspected AEs.

Logistic regression analysis showed that each additional pre-existing medical condition increased the odds of a suspected AE by approximately 2.4-fold (OR = 2.4, *p* < 0.001).

When the association between AEs and categories of pre-existing or concomitant medical conditions was analyzed, the three most common condition types were those involving the respiratory system, neurological disorders, and allergic/immunological diseases (Table 5). The full analysis is presented in Supplementary Table S2.

According to these results, immunologic disorders and allergies showed the strongest association with suspected AEs, with the largest effect size (Cramer’s V = 0.311) and an OR of 5.7. Respiratory system pathologies and neurological/neurodevelopmental disorders also showed significant associations. Some rare categories showed high point estimates, but these were based on small numbers and wide confidence intervals; therefore, they were considered exploratory.

### 3.8. Clinically Vulnerable Population

A statistically significant association was observed between belonging to a clinically vulnerable group currently targeted for COVID-19 vaccination and the occurrence of AEs, χ^2^ = 89.5, *p* < 0.001. Suspected AEs were more common in the clinically vulnerable population (70.5%) than in the non-vulnerable population (34.3%). The effect size was small to moderate (Cramer’s V = 0.21). Furthermore, belonging to a clinically vulnerable population was associated with a higher probability of a suspected AE (OR = 4.6; 95% CI: 3.3–6.4).

### 3.9. COVID-19 Infection

A statistically significant association was observed between previous COVID-19 infection and the occurrence of suspected AEs, χ^2^ = 208.1, *p* < 0.001. AEs were more common in patients with previous COVID-19 infection (69.1%) than in those without it (29.9%). The effect size was moderate (Cramer’s V = 0.320). Furthermore, previous COVID-19 infection was associated with a higher probability of a suspected AE (OR = 5.3; 95% CI: 4.1–6.7).

A summary of the univariate analysis of demographic and clinical factors and AEs is shown in Table 6.

A multivariable logistic regression model was employed to identify independent factors associated with suspected AEs. The selection of variables for the multivariate logistic regression model was based on statistical and clinical criteria, taking into account statistical significance in the bivariate analysis, the strength of the association, the precision of the confidence intervals, the effect size, and the clinical plausibility. Given that all variables retained for multivariable analysis had *p*-values < 0.001 in univariate screening, correction for multiple comparisons would not alter variable selection and priority was given to those with the strongest associations and greatest clinical relevance: weight, height, number of concomitant medications administered before vaccination, number of previous or current medical conditions, previous COVID-19 infection, or belonging to the clinically vulnerable population currently targeted for COVID-19 vaccination. Severe collinearity was identified between weight, height, and BMI, with VIF values of 57.254, 35.304, and 19.684, respectively, as well as a maximum condition number of 215.369. Weight and height were selected for the final model because BMI is not an independent measure, and in children, it must be interpreted according to age and sex. Weight and height were retained despite being collinear due to their clinical significance. Chronic treatments and polypharmacy, although they showed high odds ratios, showed a wide confidence interval and a low Cramer’s V, suggesting a less precise estimate and possible instability, as well as probable multicollinearity with other pharmacological variables (chronic treatment: 6.3% of patients; polypharmacy: 0.5% of patients, with an expected cell count below 5 in the polypharmacy contingency table). For this reason, they were excluded from the main model. The results of the initial multivariable logistic regression model are presented in Table 7.

The variables significantly associated with the occurrence of AE were height, number of medicines, number of pre-existing conditions, and previous COVID-19 infection. Weight did not reach statistical significance, although it was close to the conventional threshold of 0.05. The clinically vulnerable population showed no significant association with AE.

The final model (Table 8) was selected based on statistical significance, model parsimony, and clinical interpretability. Variables included in the final model were weight, height, number of comorbidities, number of concomitant medications, and previous SARS-CoV-2 infection.

The model was statistically significant (χ^2^ = 90.053, gl = 8, *p* < 0.001) and correctly classified 75.0% of patients.

In this adjusted model, the variables significantly associated with suspected AEs were height, number of medicines, number of pre-existing conditions, and previous COVID-19 infection. Weight did not reach statistical significance (OR = 0.98; 95% CI: 0.96–1.00; *p* = 0.087). The model was statistically significant (χ^2^ = 90.053, gl = 5, *p* < 0.001). Collinearity diagnostics showed high VIF values for weight (VIF = 57.3) and height (VIF = 35.3), consistent with the well-known biological correlation between these anthropometric measures and explaining the loss of the statistical significance of weight once height was included in the model. The final model showed good discriminative capacity, with an AUC-ROC of 0.806, a sensitivity of 52.8%, and a specificity of 88.3% at the 0.5 cut-off.

Each additional concomitant medication was associated with a 7.8-fold increase in the odds of experiencing an adverse event. The following were also observed:

- Each additional pre-existing medical condition increased the odds of AE by a factor of 1.9.
- Previous SARS-CoV-2 infection was independently associated with a 4.1-fold increase in the odds of experiencing an adverse event after vaccination.

Although weight and height were associated with suspected AEs in the univariable analysis, weight was not retained in the final multivariable model and may reflect age- or development-related differences. Given the collinearity between height and weight, height may act partly as a proxy for developmental/pubertal stage rather than as a fully independent predictor. Although the odds increase of 2.9% per centimeter (OR 1.03) appears modest, it accumulates substantially across the height range observed in this pediatric cohort, suggesting that the association is clinically relevant despite the small per-unit effect.

A sensitivity analysis was performed by re-fitting the multivariable model with the addition of “clinically vulnerable population,” “chronic treatment,” and “polypharmacy.” The magnitude and direction of the effect estimates for weight, height, number of medications, number of comorbidities, and previous COVID-19 infection remained essentially unchanged, supporting the robustness of the final model.

## 4. Discussion

This study expands upon our previous report describing the safety profile of BNT162b2 vaccination in children. While the earlier analysis [7] demonstrated that adverse events were predominantly mild and infrequent, the present study provides additional information regarding the characteristics of children more likely to experience these events. Most events included in the present analysis were mild local or systemic reactions, and serious events were extremely uncommon [7]. Therefore, the identified factors should not be interpreted as predictors of severe vaccine toxicity, but rather as variables associated with the likelihood of reporting any suspected post-vaccination AEs.

In this large real-world cohort of children aged 5–11 years, univariate findings should be considered exploratory and hypothesis-generating, as they describe crude associations without accounting for confounding or overlap between related variables. Therefore, the multivariable logistic regression model was considered the primary analysis for identifying factors independently associated with reported suspected AEs: previous SARS-CoV-2 infection, medical complexity, and concomitant medication. In contrast, age, sex, and body mass index were not significantly associated with adverse event occurrence. These findings suggest that underlying clinical characteristics may play a more important role in post-vaccination symptoms [10,11,12]. An important consideration is that the associations observed in this study may reflect both true biological reactogenicity and other reported symptoms. Children with chronic medical conditions, medication use, or clinically vulnerable status may have heightened clinical surveillance, more frequent contact with healthcare services, and parents or caregivers who are more attentive to post-vaccination symptoms. Consequently, mild or non-specific symptoms may be more likely to be noticed, remembered, or reported in these children than in otherwise healthy children. Therefore, the observed associations with comorbidity burden and concomitant medication may be best interpreted as useful clinical markers for identifying children who may benefit from closer post-vaccination monitoring, rather than as evidence of increased biological susceptibility alone.

Previous SARS-CoV-2 infection emerged as one of the strongest factors associated with reported suspected AEs. This association is biologically plausible, as previous infection may act as an immunological priming event, resulting in a more robust immune response following subsequent exposure to vaccine antigens. Studies evaluating mRNA COVID-19 vaccines have reported stronger humoral and cellular immune responses among previously infected individuals, a phenomenon that may also be accompanied by increased reactogenicity [12,13,14].

When focusing on the population for whom COVID-19 vaccination is currently recommended, our findings should be interpreted in the context of current pediatric recommendations, which prioritize vaccination in infants, children, and adolescents at increased risk of severe COVID-19, including those with chronic pulmonary disease, cardiovascular disease, obesity, immunosuppressive conditions, autoimmune diseases, neurological disorders, and other relevant comorbidities. Available safety data in children show that local and systemic reactions after COVID-19 vaccination are common, particularly after the second dose, but are generally mild to moderate, self-limited, and manageable, while serious adverse events are rarely reported [6,7]. Although our results suggest that clinical complexity may be associated with reported post-vaccination symptoms, further analyses focused on medically vulnerable pediatric subgroups are needed to better characterize their individual risk profile and to distinguish increased expected reactogenicity from clinically relevant safety concerns [11,15,16].

The association between medical history and suspected AEs may have several explanations. Some conditions, particularly allergic or immunological disorders, could plausibly be associated with heightened immune responsiveness. However, the caregivers of children with pre-existing conditions may be more likely to report mild, transient, or non-specific symptoms. Thus, comorbidity burden should be interpreted as a marker of clinical complexity associated with AE occurrence or reporting, rather than as definitive evidence of increased biological reactogenicity. From a clinical perspective, these findings reinforce the value of careful pre-vaccination assessment. In particular, healthcare professionals should document previous SARS-CoV-2 infection status, review the number and type of pre-existing medical conditions, and inquire about concomitant medications being used at the time of vaccination. Although these factors should not be considered contraindications to COVID-19 vaccination, their identification may help clinicians provide individualized counseling, set appropriate expectations regarding potential post-vaccination reactions, and determine whether closer follow-up or observation may be warranted in selected children. Such an approach may contribute to improving family reassurance, strengthening confidence in vaccination, and optimizing patient-centered vaccine care.

This may be particularly relevant in individuals with a history of allergic reactions, since early post-authorization safety data for the Pfizer-BioNTech COVID-19 vaccine showed that most anaphylaxis cases occurred in individuals with a documented history of allergies or previous allergic reactions, although anaphylaxis remained rare and most patients recovered or were discharged. Current safety recommendations therefore emphasize pre-vaccination screening, availability of appropriate treatment for anaphylaxis, and post-vaccination observation according to the individual allergic history. Thus, the high odds ratio observed for medical history in our study should not be interpreted as a contraindication to vaccination, but rather as a signal that patients with relevant clinical backgrounds may benefit from careful pre-vaccination assessment and appropriate post-vaccination monitoring [13,15,16,17].

The strong association observed between concomitant medication use and reported suspected AEs should also be interpreted cautiously, but it is biologically and clinically plausible [18]. In pediatric populations, the number of concomitant drugs has been associated with an increased prevalence of AEs, even after adjusting for disease categories, suggesting that polypharmacy may contribute to AE risk. Medication burden may not represent a direct causal determinant of vaccine reactogenicity; instead, it may represent a proxy for underlying disease severity, acute intercurrent illness, healthcare contact, and increased parental vigilance. The decrease in the odds ratio from 15.1 for any concomitant medication to 5.1 for chronic medication may only be explained by the broader clinical meaning of the former variable. Concomitant medication, including both acute and chronic treatments, may capture acute intercurrent conditions or increased healthcare contact around vaccination, which could be more strongly associated with adverse event occurrence or reporting. In contrast, chronic medication more specifically reflects a stable underlying disease, resulting in a lower but still clinically relevant association [18].

Age was not significantly associated with adverse events in our study. This finding is consistent with clinical trials of BNT162b2 in children aged 5–11 years, and other studies conducted in adolescents vaccinated with BNT162b2 or mRNA-1273 have shown a similar safety profile, suggesting that age alone may not be a major determinant of post-vaccination adverse event occurrence within pediatric populations. Rather, individual clinical background, previous SARS-CoV-2 exposure, and immune responsiveness may be more relevant than age itself [10,11,12,13,19,20,21].

BMI was not significantly associated with AEs in our cohort, suggesting that it may not be a sufficiently representative anthropometric marker for assessing vaccine-related risk in children. This is plausible because BMI does not adequately reflect body composition, pubertal status, metabolic health, or immune function. The factor that may be more relevant is excess weight itself; however, current evidence does not allow us to determine whether overweight is associated with a higher risk of adverse events or rather with differences in vaccine immunogenicity [10,22], potentially due to altered immune responses or a dilution effect. Future studies should therefore evaluate overweight and obesity according to developmental stage to better clarify their influence on vaccine safety and immune response in pediatric populations.

Sex was also not significantly associated with AEs in our analysis. Although sex-based differences in immune responses to vaccines have been described, with females often showing stronger humoral and cellular responses and, in some studies, more frequent or more severe post-vaccination reactions, these differences are not universal. Available evidence suggests that sex-related differences may vary according to age, vaccine type, previous infection status, hormonal and genetic factors, and underlying comorbidity profile [10,12,23]. As our cohort included children aged 5–11 years, before full pubertal maturation, sex-based differences in reactogenicity may be less pronounced than in adolescents or adults.

From a clinical perspective, our findings emphasize the value of a detailed pre-vaccination assessment, particularly in children with previous SARS-CoV-2 infection, chronic medical conditions, or ongoing pharmacological treatment. Identifying these characteristics may help healthcare professionals provide individualized counseling and anticipatory guidance regarding expected post-vaccination reactions while reinforcing the overall favorable safety profile of COVID-19 vaccines.

Overall, our results suggest that clinical complexity, particularly comorbidity burden and concomitant medication use, may be more relevant than demographic variables such as age, sex, or BMI in predicting AEs after COVID-19 vaccination. Future studies should include larger pediatric and clinically vulnerable populations; stratified analyses by type of comorbidity, allergic history, previous SARS-CoV-2 infection, and medication burden; and multivariable models capable of distinguishing independent predictors from markers of underlying disease severity [10,11,12,13,15,16,18].

### Limitations and Strengths

The present work is a secondary analysis derived from a cohort that has previously been used to evaluate the overall safety profile of pediatric COVID-19 vaccination [7]. However, the research question, statistical approach, and study outcomes addressed here differ substantially from those reported previously, as this study focuses on risk factors associated with adverse event occurrence rather than on adverse event incidence and characterization.

Several limitations should be acknowledged. First, AEs were reported by parents or guardians and may therefore be subject to recall bias and reporting bias. Second, the study was conducted in a single geographical region, potentially limiting the generalizability of the findings. Third, a substantial proportion of vaccinated children could not be included because of unavailable contact information, unsuccessful follow-up, lack of consent, or insufficient clinical data, introducing the possibility of selection bias. Finally, some clinical subgroups were small, resulting in wide confidence intervals and less precise effect estimates. The predominance of participants without comorbidities may have influenced the overall prevalence estimates; however, it is unlikely to have substantially affected the observed associations, given the consistency of the multivariable analyses and the adequate representation of individuals with comorbid conditions. Nevertheless, further studies specifically focused on vulnerable populations are needed to confirm the magnitude and generalizability of these findings within these groups.

Another limitation is the difficulty in distinguishing true biological reactogenicity from other symptoms reported. Because AEs were identified through parental or guardian reports and medical record review, children with chronic conditions, medication use, or vulnerable status may have been subject to greater parental vigilance, healthcare contact, and clinical surveillance. This may have increased the likelihood of detecting or reporting mild or non-specific symptoms. It is important to mention the possibility that predictors of reactogenicity differ by dose, and that this should be explored in future studies.

Strengths include the large real-world pediatric cohort, the systematic follow-up, the detailed review of electronic health records, and the comprehensive evaluation of demographic and clinical predictors.

## 5. Conclusions

In this large real-world cohort of children aged 5–11 years vaccinated against COVID-19, previous SARS-CoV-2 infection, greater comorbidity burden, and concomitant medication use were associated with the occurrence of adverse events following immunization. These findings suggest that underlying clinical complexity may be an important determinant of vaccine reactogenicity and highlight the value of individualized risk assessment in pediatric vaccination programs. Further studies are needed to better characterize the mechanisms underlying these associations.

## Figures and Tables

**Table 1:** Demographic and clinical characteristics of the patients included in the study.

Variable	Mean	Standard Deviation	Minimum	Maximum
Age (months)	109.5	25.9	60	144
Weight (Kg)	31.0	9.2	13	82
Height (cm)	132.0	13.67	90	180
Body Mass Index	17.5	2.80	9.5	34.7
**Variable**			**Frequency (%)**	
Sex	Female		969 (47.8)	
	Male		1057 (52.2)	
Medical History	No		1475 (72.8)	
	Yes		551 (27.2)	
Concurrent Medication	No		1786 (88.2)	
	Yes		240 (11.8)	

**Table 2:** Frequency of suspected adverse events (AEs) according to the number of concomitant drugs.

		Patients with Suspected AEs Among Non-Exposed Individuals, N/N (%)	Patients with Suspected AEs Among Exposed Individuals, N/N (%)	Total (n = 2026)
Number of concomitant drugs	0	1235 (69.2%)	551 (30.8%)	1786 (100%)
	1	30 (14.4%)	172 (85.6%)	202 (100%)
	2	1 (3.6%)	27 (96.4%)	28 (100%)
	3	1 (14.3%)	6 (85.7%)	7 (100%)
	4	0 (0%)	3 (100%)	3 (100%)
	total	1267	759	2026

Patients without AEs received an average of 0.03 drugs, compared with 0.34 in the AEs group. This association remained statistically significant (OR = 11.3; *p* < 0.001).

**Table 3:** Association between exposure to medications by ATC first-level classification and suspected adverse drug reactions (AEs).

ATC Level 1 Drug Class	Patients with Suspected AEs Among Non-Exposed Individuals, N/N (%)	Patients with Suspected AEs Among Exposed Individuals, N/N (%)	Odds Ratio	95% Confidence Interval	*p*-Value
Blood and blood forming organs	756/2023 (37.4%)	3/3 (100%)	No estimable	—	0.052 *
Musculoskeletal system	732/1991 (36.8%)	27/35 (77.1%)	5.8	2.6–12.8	<0.001
Nervous system	637/1886 (33.8%)	121/140 (86.4%)	12.5	7.6–20.4	<0.001
Respiratory system	708/1973 (35.9%)	50/53 (94.3%)	29.7	9.2–95.6	<0.001

* *p*-value based on Fisher’s test. Estimates for categories with very small numbers of exposed participants should be interpreted with caution.

**Table 4:** Frequency of suspected adverse events (AEs) according to the number of medical antecedents.

		Frequency of Patients Without Suspected AEs (%)	Frequency of Patients with Suspected AEs (%)	Total
Number of medical antecedents	0	1087 (73.7%)	389 (26.3%)	1476 (100%)
	1	112 (34.3%)	214 (65.7%)	326 (100%)
	2	46 (31.1%)	102 (68.9%)	148 (100%)
	3	13 (30.2%)	30 (69.8%)	43 (100%)
	4	7 (29.2%)	17 (70.8%)	24 (100%)
	5	2 (50%)	2 (50%)	4 (100%)
	6	0 (0.0%)	2 (100%)	2 (100%)
	7	0 (0.0%)	3 (100%)	3 (100%)
	Total	1267	759	2026

Χ^2^ = 288.2, *p* < 0.001.

**Table 5:** Association between medical antecedent and suspected adverse events (AEs).

Medical History	Patients with Suspected AEs Among Non-Exposed Individuals, N/N (%)	Patients with Suspected AEs Among Exposed Individuals, N/N (%)	Odds Ratio	95% Confidence Interval	*p*-Value
Perinatal history and congenital malformations	737/1986 (37.1%)	23/40 (57.5%)	2.3	1.2–4.3	0.008
Respiratory system	688/1922 (35.8%)	71/104 (68.3%)	3.9	2.5–5.8	<0.001
Neurology and neurodevelopment	697/1941 (35.9%)	62/85 (72.9%)	4.8	3.0–7.8	<0.001
Metabolic and endocrine disorders	728/1990 (36.6%)	30/36 (83.3%)	8.6	3.6–20.8	<0.001
Immunology and allergy	526/1702 (30.9%)	233/324 (71.9%)	5.7	4.4–7.4	<0.001
Gastroenterology	739/1999 (37.0%)	19/27 (70.4%)	4.0	1.8–9.3	<0.001

Estimates for categories with very small numbers of exposed participants should be interpreted with caution.

**Table 6:** Univariate analysis of demographic and clinical factors associated with suspected adverse events (AEs).

Variable	χ^2^/Mean Difference	*p*-Value	95% Confidence Interval
Age	−0.6	0.591	−2.8 to 1.6
Weight (kg)	−2.9	<0.001	−3.8 to −2.1
Height (cm)	−5.4	<0.001	−6.6 to −4.1
Body Mass Index	−0.2	<0.185	−0.7 to 0.1
Concomitant Medication	286.1	<0.001	15.1 (10.2–22.4)
Chronic Treatments	74.0	<0.001	5.1 (3.4–7.6)
Polymedication	11.8	<0.001	15.2 (1.9–120.1)
Medical Antecedents	288.2	<0.001	5.8 (4.7–7.1)
Vulnerable Population	89.5	<0.001	4.6 (3.3–6.4)
COVID-19 Infection	208.1	<0.001	5.3 (4.1–6.7)

**Table 7:** Initial multivariable regression model of factors associated with COVID-19 vaccine-related adverse events.

Variable	B	Standard Error	Wald	GL	*p*-Value	Odds Ratio	Inferior 95% Confidence Interval	Superior 95% Confidence Interval
Weight	−0.018	0.011	2.757	1	0.097	0.98	0.96	1.00
Height	0.028	0.007	16.007	1	<0.001	1.03	1.01	1.04
Number of concomitant drugs	2.054	0.203	102.217	1	<0.001	7.80	5.24	11.62
Number of medical antecedents	0.645	0.082	61.534	1	<0.001	1.91	1.62	2.24
COVID-19 infection	1.409	0.134	110.313	1	<0.001	4.09	3.15	5.32
Vulnerable population	−0.137	0.241	0.322	1	0.570	0.87	0.54	1.40
Constant	−4.530	0.698	42.140	1	<0.001	0.01	—	—

**Table 8:** Final multivariable regression model of factors associated with COVID-19 vaccine-related adverse events.

Variable	B	Standard Error	Wald	GL	*p*-Value	Odds Ratio	Inferior 95% Confidence Interval	Superior 95% Confidence Interval
Weight	−0.018	0.011	2.927	1	0.087	0.98	0.96	1.00
Height	0.029	0.007	16.318	1	<0.001	1.03	1.01	1.04
Number of concomitant drugs	2.050	0.203	101.823	1	<0.001	7.77	5.22	11.56
Number of medical antecedents	0.622	0.071	76.246	1	<0.001	1.86	1.62	2.14
COVID-19 infection	1.405	0.134	110.000	1	<0.001	4.07	3.13	5.30
Constant	−4.545	0.697	42.489	1	<0.001	0.01	—	—

## Data Availability

The data presented in this study are available on request from the corresponding author due to ethical reasons.

## Supplementary Materials

The following supporting information can be downloaded at: https://www.mdpi.com/article/10.3390/children13091160/s1, Table S1: Association between exposure to medications by ATC first-level classification and suspected adverse drug reactions (AEs); Table S2: Association between medical antecedent and suspected adverse events (AEs).

## Abbreviations

ACIP	Advisory Committee on Immunization Practices
AE	Adverse Event
AEs	Adverse Events
ATC	Anatomical Therapeutic Chemical Classification System
BMI	Body Mass Index
BNT162b2	Pfizer-BioNTech COVID-19 mRNA vaccine
ACIP	Advisory Committee on Vaccines of the Spanish Association of Pediatrics
CDC	Centers for Disease Control and Prevention
COVID-19	Coronavirus Disease 2019
mRNA	Messenger Ribonucleic Acid
RNA	Ribonucleic Acid
SARS-COV-2	Severe Acute Respiratory Syndrome Coronavirus 2
WHO	World Health Organization

## References

[1] Iofrío de Arce A. (2025). Estado actual de la vacunación pediátrica contra la COVID-19. Pediatr. Integral.

[2] Comisión de Salud Pública, Consejo Interterritorial del Sistema Nacional de Salud (2025). Recomendaciones de Vacunación Frente a COVID-19. 2025–2026, España. https://www.sanidad.gob.es/areas/promocionPrevencion/vacunaciones/gripe_covid19/docs/recomendacionesVacunacionCovid19_2025_2006.pdf.

[3] World Health Organization (2023). Statement on the Fifteenth Meeting of the IHR (2005) Emergency Committee on the COVID-19 Pandemic. https://www.who.int/news/item/05-05-2023-statement-on-the-fifteenth-meeting-of-the-international-health-regulations-(2005)-emergency-committee-regarding-the-coronavirus-disease-(covid-19)-pandemic.

[4] Department of Health, Human Services (2025). United States Government. ACIP Recommends COVID-19 Immunization Based on Individual Decision-Making. https://www.hhs.gov/press-room/acip-recommends-covid19-vaccination-individual-decision-making.html.

[5] Cuadro de Mando Resumen de Datos de Vacunación. Ministerio de Sanidad Española. https://www.sanidad.gob.es/areas/alertasEmergenciasSanitarias/alertasActuales/nCov/pbiVacunacion.htm.

[6] Hause A.M., Baggs J., Marquez P., Myers T.R., Gee J., Su J.R., Zhang B., Thompson D., Shimabukuro T.T., Shay D.K. (2021). COVID-19 vaccine safety in children aged 5–11 years—United States, November 3–December 19, 2021. MMWR Morb. Mortal. Wkly. Rep..

[7] Ángeles Rivas Paterna M., Pérez Ingidua C., Rivas Paterna A.B., Ortega Martínez D., Aleo Luján E., Vargas Castrillón E. (2026). Reacciones adversas a la vacuna COVID-19 en población pediátrica en España. An. Pediatr..

[8] Harris P.A., Taylor R., Thielke R., Payne J., Gonzalez N., Conde J.G. (2009). Research electronic data capture (REDCap): A metadata-driven methodology and workflow process for providing translational research informatics support. J. Biomed. Inform..

[9] World Health Organization (1972). International Drug Monitoring: The Role of National Centres. Report of a WHO Meeting.

[10] Zimmermann P., Curtis N. (2019). Factors That Influence the Immune Response to Vaccination. Clin. Microbiol. Rev..

[11] Hause A.M., Shay D.K., Klein N.P., Abara W.E., Baggs J., Cortese M.M., Fireman B., Gee J., Glanz J.M., Goddard K. (2022). Safety of COVID-19 Vaccination in United States Children Ages 5 to 11 Years. Pediatrics.

[12] Sáez-Peñataro J., Calvo G., Bascuas J., Mosquera M.M., Marcos M.Á., Egri N., Torres F. (2024). Association between Reactogenicity and Immunogenicity in a Vaccinated Cohort with Two mRNA SARS-CoV-2 Vaccines at a High-Complexity Reference Hospital: A Post Hoc Analysis on Immunology Aspects of a Prospective Cohort Study. Vaccines.

[13] Lee J., Woodruff M.C., Kim E.H., Nam J.-H. (2023). Knife’s Edge: Balancing Immunogenicity and Reactogenicity in mRNA Vaccines. Exp. Mol. Med..

[14] Menni C., Klaser K., May A., Polidori L., Capdevila J., Louca P., Sudre C.H., Nguyen L.H., Drew D.A., Merino J. (2021). Vaccine side-effects and SARS-CoV-2 infection after vaccination in users of the COVID Symptom Study app in the UK: A prospective observational study. Lancet Infect. Dis..

[15] Committee on Infectious Diseases (2025). Recommendations for COVID-19 Vaccines in Infants, Children, and Adolescents. Pediatrics.

[16] European Medicines Agency (EMA) Comirnaty. European Medicines Agency. https://www.ema.europa.eu/en/medicines/human/EPAR/comirnaty.

[17] Shimabukuro T., Nair N. (2021). Allergic Reactions Including Anaphylaxis After Receipt of the First Dose of Pfizer-BioNTech COVID-19 Vaccine. JAMA.

[18] Sugioka M., Tachi T., Mizui T., Koyama A., Murayama A., Katsuno H., Matsuyama T., Aoyama S., Osawa T., Noguchi Y. (2020). Effects of the number of drugs used on the prevalence of adverse drug reactions in children. Sci. Rep..

[19] Walter E.B., Talaat K.R., Sabharwal C., Gurtman A., Lockhart S., Paulsen G.C., Barnett E.D., Muñoz F.M., Maldonado Y., Pahud B.A. (2022). Evaluation of the BNT162b2 COVID-19 Vaccine in Children 5 to 11 Years of Age. N Engl. J. Med..

[20] Frenck R.W., Klein N.P., Kitchin N., Gurtman A., Absalon J., Lockhart S., Perez J.L., Walter E.B., Senders S., Bailey R. (2021). C4591001 Clinical Trial Group. Safety, Immunogenicity, and Efficacy of the BNT162b2 COVID-19 Vaccine in Adolescents. N. Engl. J. Med..

[21] Ali K., Berman G., Zhou H., Deng W., Faughnan V., Coronado-Voges M., Ding B., Dooley J., Girard B., Hillebrand W. (2021). Evaluation of mRNA-1273 SARS-CoV-2 Vaccine in Adolescents. N. Engl. J. Med..

[22] Centers for Disease Control and Prevention About Child & Teen BMI. U.S. Department of Health and Human Services. https://www.cdc.gov/healthyweight/assessing/bmi/childrens_bmi/about_childrens_bmi.html.

[23] Klein S.L., Marriott I., Fish E.N. (2015). Sex-based differences in immune function and responses to vaccination. Trans. R. Soc. Trop. Med. Hyg..

