# Supplementary material for: Risk Factors Associated with Adverse Events Following COVID-19 Vaccination in Children Aged 5–11 Years: A Real-World Observational Study
Source: Children (Basel). 2026 Aug 28;13(9):1160. doi: 10.3390/children13091160 (PMC13605865; doi:10.3390/children13091160)
Supplement: Supplementary file 1 [file children-13-01160-s001.zip › children-4480711-supplementary.pdf]

Supplementary Table S1. Association between exposure to medications by ATC first-level classification and suspected adverse drug reactions (AEs).

ATC level 1 Drug Class	Patients with suspected AEs among non-exposed individuals, N/N (%)	Patients with suspected AEs among exposed individuals, N/N (%)	Odds Ratio	95% Confidence Interval	p-value
**Alimentary tract and metabolism**	751/2018 (37.2%)	8/8 (100%)	No estimable	—	<0.001
**Blood and blood forming organs**	756/2023 (37.4%)	3/3 (100%)	No estimable	—	0.052*
**Cardiovascular system**	758/2024 (37.5%)	1/2 (50,0%)	1.7	0.1–26.7	1.000
**Genito-urinary system and sex hormones**	759/2025 (37.5%)	0/1 (0%)	No estimable	—	1.000
**Systemic hormonal preparations**	753/2020 (37.3%)	5/6 (83,3%)	8.4	0.9–71.9	0.030
**Antineoplastic and immunomodulating agents**	755/2022 (37.3%)	4/4 (100%)	No estimable	—	0.020
**Musculo-skeletal system**	732/1991 (36.8%)	27/35 (77.1%)	5.8	2.6–12.8	<0.001
**Nervous system**	637/1886 (33.8%)	121/140(86.4%)	12.5	7.6–20.4	<0.001
**Respiratory system**	708/1973 (35.9%)	50/53 (94.3%)	29.7	9.2–95.6	<0.001
**Sensory organs**	756/2023 (37.4%)	3/3 (100%)	No estimable	—	0.052*
**Various**	755/2022 (37.3%)	4/4 (100%)	No estimable	—	0.020

* p-value base on Fisher test.

Supplementary Table S2. Association between medical antecedent and suspected adverse events (AEs).

Medical History	Patients with suspected AEs among non-exposed individuals, N/N (%)	Patients with suspected AEs among exposed individuals, N/N (%)	Odds Ratio	95% Confidence Interval	p-value
**Perinatal history and congenital malformations**	737/1986 (37.1%)	23/40 (57.5%)	2.3	1.2–4.3	0.008
**Respiratory system**	688/1922 (35.8%)	71/104 (68.3%)	3.9	2.5–5.8	<0.001
**Cardiovascular system**	744/2001 (37.2%)	15/25 (60.0%)	2.5	1.1–5.7	0.019
**Neurology and neurodevelopment**	697/1941 (35.9%)	62/85 (72.9%)	4.8	3.0–7.8	<0.001
**Metabolic and endocrine disorders**	728/1990 (36.6%)	30/36 (83.3%)	8.6	3.6–20.8	<0.001
**Immunology and allergy**	526/1702 (30.9%)	233/324 (71.9%)	5.7	4.4–7.4	<0.001
**Infectious diseases**	750/2010 (37.3%)	9/16 (56.3%)	2.2	0.8–5.8	0.119
**Gastroenterology**	739/1999 (37.0%)	19/27 (70.4%)	4.0	1.8–9.3	<0.001
**Dermatology**	749/2015 (37.2%)	9/11 (81.8%)	7.6	1.6–35.2	0.002
**Previous surgeries**	724/1963 (36.9%)	34/63 (54.0%)	2.0	1.2–3.3	0.006
**Mental health**	751/2017 (37.2%)	8/9 (88.9%)	13.5	1.7–108.0	0.001
**Traumatology and musculoskeletal system**	755/2016 (37.4%)	6/10 (60.0%)	2.5	0.7–8.9	0.140
**Eyes and related organs**	745/2012 (37.1%)	13/14 (92.9%)	22.1	2.9–1689.0	<0.001
**Otolaringological diseases**	751/2014 (37.3%)	7/12 (58.3%)	2.3	0.7–7.4	0.134
**Genitourinary system**	750/1995 (37.6%)	9/31 (29.0%)	0.7	0.3–1.5	0.328
**Haematology and coagulation**	754/2017 (37.4%)	5/9 (55.6%)	2.1	0.7–7.8	0.261

Estimates for categories with very small numbers of exposed participants should be interpreted with caution.
